# Collectanea Medica: Consisting of Anecdotes, Facts, Extracts, Illustrations, &c.

**Published:** 1819-11

**Authors:** 


### COLLECTANEA MEDICA:

CONSISTING OF

### ANECDOTES, FACTS, EXTRACTS, ILLUSTRATIONS, &c.

*Relating to the History or the Art of Medicine, and the Auxiliary Sciences.*

Floriferis ut apes in saltibus omnia libant,

Omnia nos itidem depascimur aurea dicta.

### *On the Departments and Periodical Actions of the Glands.*

[Bordeu, *Reckérches sur les Glandes,* § cxix.]

WE shall term the *department** of a gland all that part of the system which enters into a sort of action when the gland acts: when these organs dispose themselves for the exercise of their functions, it is certain that they agitate more or less the neighbouring parts.

There are some glands which, when in action, suspend other functions, so that we may say, that, in certain respects, the suppression of those functions is comprised in the *department* of the gland which suspends them.

We even find some glandular bodies which augment, when they take on a certain kind of action, the motion of the heart, and which give shocks, more or less regular, to the whole nervous system.

We shall endeavour to elucidate these truths, which have been only perceived in a general manner. It will be necessary to determine the department of each gland, to know which are those that may act at the same time with it, and which are *congeneras;* and to ascertain how long they are engaged in action. Let us see, if what we are about to adduce will not throw some light on this matter.

The glands act to effect their excretion; there are periods when they are not in action; their action is, as it were, periodical:—these truths have been demonstrated in a former part of this dissertation.

Are not these periods marked and fixed? and what are the variations that may take place in them? This problem, and many others that might be proposed on this matter, are not easy of solution: we have not yet acquired the necessary materials. Let us see, however, if it is not possible to say something more determinate on the departments of the glands, and on their periodical actions.

*An examination of what passes in the Liver.*—Let us first speak of the gall-bladder; and, without stopping to consider its true position in all subjects, let us only examine what authors have said on the manner in which it evacuates its contents.

We find some who state that it is compressed by the stomach: the

* We now call it the *sympathetic relations* of an organ; but the word sympathy was not used in medical language at the time of Bordeu.—Edit.

most modern authors render it subject to the action of the duodenum: but we do not fear to assert, that, if there were only these pretended causes for its evacuation, that this would never take place.

1°. The stomach does not touch it in hardly any subject; 2°, the duodenum touches it only in a few; 3°, every person may prove that compressing it in the dead body, and even with force, does not empty it: it must be very full for compression to produce a flow of bile from it; 4°, the colon constantly touches the gall-bladder,—but will it be said that it is to compress it? It must only empty itself, then, when the colon is full: which is an absurd supposition. Let us conclude from these proofs, without entering into long discussions, that the gall-bladder must be emptied by some other power than that of the compression of the surrounding parts.* We must not think that compression has nothing to do with it; but it is necessary that the gall-bladder should itself enter into action, and make an effort which renders it as if it were full. It is like the urinary bladder, like the rectum, the stomach, and the intestines: these reservoirs empty themselves by their own powers, and by the effort of the parts in their vicinity.

The gall-bladder is, without doubt, irritated by the matters which are in the duodenum, and which cause an appropriate irritation of the biliary duct, and the nerves of these parts; perhaps, indeed, the gall-bladder enters into action without being thus irritated.

The liver should certainly be regarded as one of the glands which we have termed active, and which have a particular motion for secretion and for excretion.

This glandular viscus is constantly agitated and irritated by the diaphragm; it is perpetually rising and falling; so it is probable it is always in some degree engaged in secretion: but is this secretion as continual as that of the saliva?

There is some period in which the secretion is much augmented; and this is apparently, under ordinary circumstances, when digestion is going on in the duodenum.

Habit, apparently, prevents our feeling the motions of the liver; inspection of living animals does not teach us any thing positive; we have, however, convincing proofs of such a mode of action of the liver: disease, and various preternatural states, furnish them.

Indeed, there are some persons, who, an hour or two after eating, become extremely yellow: they remark that this constantly happens during the period which they call the time of digestion, or when the aliment which has sojourned in the stomach passes into the duodenum.

---

* We should have observed on first adducing these extracts, for the convenience of our less well-informed readers, that the arguments in them are expressly directed against the doctrine of Böerhaave, whose sectaries at that time were the medical heroes of Europe; and, indeed, not less celebrated in almost every part of the civilized world. The emperor of China, on writing to their master, addressed his letter, To Böerhaave, in *Europe*. Bordeu, in his Thesis, produced at the age of twenty-one, commenced his attack on that doctrine, and displayed some of those conceptions that were the foundation for the revolution that has since ensued.—Edit.

Whence comes this bile which is thus shown in the blood? Is it not evident that it reflows from the liver, and that it is impelled by the action of this viscus: this action is too forcible, and the vibrations are ill directed. But, do not these periodical returns prove two things? One, that the liver has, like all other *active* glands, a particular action for the discharge of the fluids which it separates, or for their separation; and the other, that this action is renewed, or augmented, or comes on, precisely during the time of taking food.

Those who are subject to certain colics, which have a great relation to the liver, and which are often the consequences of stones contained in the gall-bladder, have they not paroxysms, or more severe attacks, of these colics during the process of digestion? Do they not feel constrictions, stiches, and drawing sensations, in the hypochondres?

We might also relate what takes place in melancholic patients, in whom it is easy to perceive that the stiches, tremulous sensations, and dull pains, of which they complain, in the right side, have their seat in the liver: let us, however, only speak of those who are subject to hæmorrhoids. When the flux of blood is about to happen, the patients feel painful stiches and sharp darting sensations, which commence towards the right hypochondre, and which proceed in a downward direction to the left side. Is it possible to doubt that the liver, and the parts connected with it, are not in action in these cases?

Lastly, those who suffer schirrous tumors and chronic abscesses in the liver, have pains, in a manner periodical, in the hypochondre. These pains are more or less fixed, and they are experienced more at one time than at another; and ordinarily they are increased during the process of digestion.

The patients in question are also subject to darting pains in the whole of the right side, about the neck and face, and to numbness of the arm and lower extremity of the same side: after these darting pains have continued for some time, swelling comes on in the right side, the face, the arm, the leg, the hypochondre, &c.

All these observations, which daily experience confirms, demonstrate, it appears to me, that the liver has a peculiar action, and that it exerts it especially during the time of digestion. They also show that there are parts dependant, in a manner, on the liver; since, when that organ is diseased, they experience the effects of it: these are in the department of the liver.

*An examination of what passes in the Spleen.*—Whatever may be the principal use of the spleen, we shall demonstrate that it has a peculiar action, and that it exerts it at marked periods; and, lastly, that it has a very extensive department.

Let us first make use of the remarks of M. Duvernoy and those of M. Lieutaud, which we have in part confirmed. The spleen is of a different colour, and different in size, in dogs, when they are opened whilst digestion is going on, and when it is completed: if the stomach be full, the spleen is *folded up*, white, and hard; on the contrary, it is more soft, more red, and larger, when the stomach is not full. These observations cannot be disputed.

We do not however believe, as it has been done, that the diminution in the bulk of the spleen arises from the compression made on it by the stomach; it appears, that there is no state in which the spleen is not harder than the stomach. Although there were not many reasons which we will not here detail, this remark alone is sufficient to prove that the spleen is never compressed by means of the stomach.

How do the changes we have mentioned arise then? The following observations will perhaps explain them. The stomach admits blood much more easily when it is full than when it is empty; the circulation is carried on better in this organ when it is distended than when it is empty: now, since the spleen and the stomach have arteries from one trunk, it follows, that, when the blood flows more readily into the stomach, the spleen will receive less of that fluid.

We must also add, that the spleen contracts itself by its own power, for the want of blood would not render it hard: it has its peculiar action; and this action, which diminishes the volume of this viscus, causes the blood to go in greater quantity to the stomach, to the pancreas, and apparently to the liver itself; and this precisely when secretion should be augmented in all these viscera.

The spleen may then be regarded as an organ, which, when it is full, diminishes the quantity of fluids in some other viscera; and which, when it contracts or empties itself, augments the quantity of those fluids. We might, in this sense, consider the spleen, in certain respects, as a sort of reservoir, which we have already remarked: we do not, however, mean to say, that this is the principal and the only use of it.

Whatever it may be, the action of the spleen shows itself still better in what we perceive in some diseases. Those who have tumors and suppurations in this viscus, experience in the left side similar disorders to those which are found in the right when the liver is morbidly affected,—as darting pains, flutterings, distensions, &c.

We have seen a man who had a schirrous tumor in the spleen, who ordinarily, towards the hours of eight or nine in the morning, had the left foot almost totally benumbed; and the cheek of the same side cold as marble, whilst the right was very hot.

We should never end, were we to treat of all the pains in the head, fluxions of the left eye, pains in the ear of the same side, cramps, and many other phenomena, observed on the left side of those who have disease of the spleen.

This, then, is another viscus, apparently glandular, that has its action and its department, and performs its functions after certain intervals.

This is not the place to examine whence these communications, or these relations of one part to another, arise; and how the shocks and thrills extend from the affected viscus, as from a centre.

It is sufficient, that what we have advanced is founded on incontestible observations, and which those who regard patients with some attention will not deny.

### *History of the Case of a Woman, whose Skin became Black consequently to a violent Moral Emotion.**

Mary Agnes Letellier, 75 years of age, born at Troyes in Champagne, of white parents, and herself perfectly white until the period of the accident we are about to describe, of a feeble constitution, had menstruated irregularly, and had, during her life, experienced a variety of maladies; she had aborted twice, experienced an inguinal hernia, had luxated the femur, and been affected with various attacks of inflammation of the thoracic and abdominal viscera.

Towards the commencement of the French revolution, she was charged with having uttered some benevolent expressions respecting the king, imprisoned, and condemned to death for this imputed crime. The fatal lantern, the instrument of her destruction, was soon let down before her. At this terrific object, menstruation, which she then experienced, immediately became suppressed. Her execution was suspended by the intercession of some person of high authority. Soon afterwards (she said a few days) her skin assumed the colour it ever afterwards preserved. The hue of her countenance became like that of the less dark negroes, but her features did not suffer any alteration of form. The colour became deeper in the neck and shoulders; the chest was of nearly the same degree of blackness as the face. The skin covering the abdomen and lower extremities was not so dark as that of the chest. What was very remarkable, was the marbled appearance on the limbs, arising from the interspersion of white spots: this is also remarked in the American blacks. The joints of the fingers were blacker than the other part of them; the soles of the feet, palms of the hands, and folds of the inguinal region, were the least coloured parts of the whole body. From the time of this afflicting event, the health of the patient became more and more languishing; she became subject to beating in the head, with sense of oppression and general uneasiness. She at length fell a victim to chronic inflammation of the intestines, in the month of April 1819. During the whole time of the disease the skin was supple, and even oily; cutaneous perspiration did not appear to be either diminished or increased; the scales of which the cutaneous tissue is composed were larger than ordinary.

A vesicatory was applied in the course of the disease, and the epidermis raised by its action was perfectly transparent. The subjacent tissue was brownish.

*Examination of the dead body.—External appearance.* The colour of the skin had not suffered any change. *The mucous tissue, separated from the dermoid and epidermoid texture by maceration, was brown, like that of negroes.* All the other tissues had preserved the colour natural to whites.

*The head.*—The brain was perfectly healthy in appearance, as were its envelopments; there was rather an abundant effusion of serum into the ventricles.

*The chest.*—There were old adhesions of the pleura, but no apparent alteration of the parenchymatous structure of the lungs. The peri-

* Related by M. Rostan, in the *Nouveau Journal de Medicine,* Mai 1819.

cardium was about a line in thickness, and of so cartilaginous and dense a consistence, that it was impossible to distinguish whether or not it was formed of super-imposed layers. The heart was voluminous, the sides of the ventricles very thick and firm; but all the openings were free, and all the cavities in their natural state. No unusual opening, no extraordinary communication, was remarked.

*The abdomen.*—The mucous membrane of the stomach was slightly injected with blood, in dispersed patches. This organ contained a liquid, rather abundant in quantity, of an opake-yellow colour, much resembling the yolk of an egg dissolved in warm water. The small intestines were much injected, and presented unequivocal traces of inflammation. They contained similar matter to that in the stomach, only it was of greater consistence.

*The liver* was perfectly healthy, and it did not appear ever to have been diseased. The passages of the gall-bladder and biliary ducts were not at all obstructed, and the contained bile passed with the greatest readiness into the duodenum. The spleen, pancreas, kidneys, and bladder, did not show any vestige of alteration from the natural state.

---

### *Observations on the Plague.* By Dr. James Grey Jackson.

His Majesty's ship which was lying in the port of Alexandria when Colonel Fitzclarence passed through Egypt from India, on his way to England, convoyed to Tangier a vessel which had on-board two of the sons of Muley Soliman emperor of Morocco. On their arrival at Tangier, the princes immediately landed, and proceeded to their father at Fas; but it was discovered by the governor, or alkaid, of Tangier, that, during the passage, some persons had died; and, accordingly, the alkaid would not suffer any of the passengers to land, except the princes, until he should have received orders from the emperor how to act: he accordingly wrote to Fas for the imperial orders, and in the mean time the princes arrived, and presented themselves to the emperor. The latter wrote to the alkaid, that, as the princes had been suffered to land, it would be unjust to prohibit the other passengers from coming ashore also: he therefore ordered the alkaid to suffer all the passengers, together with their baggage, to be landed; and soon afterwards the plague appeared at Fas and at Tangier. Thus, the contagion which is now ravaging West Barbary, was imported from Egypt. It does not appear that the mortality is, or has been, during its acme at Fas, any thing comparable to what it was during the plague that ravaged this country in 1799, and which carried off more than two-thirds of the population of the empire.

Whence proceeds this difference? Is it a different species of plague, and not so deadly a contagion? or is it because the remedy of *olive oil*, applied and recommended generally by me, and by some other Europeans, during the plague of 1799, is now made public, and generally administered? This is an inquiry well deserving the attention of scientific men; and his Majesty's ministers might procure the information from the British consul at Tangier, or from the governor of Gibraltar. Perhaps the truth is, that the contagion is of a more mild character.

With regard to the remedy of olive oil applied *internally*,* I should myself be disposed to doubt its efficacy, unless M. Colaco, the Portuguese consul at l'Araich, is competent to declare, from his own knowledge and experience, that this remedy has been administered effectually by him to persons having the plague, who did not also use the friction with oil. I say, till this can be ascertained, I think the remedy of oil applied externally should not be forsaken; as it has been proved, during the plague in Africa in 1799, to be infallible, and therefore indispensable to people whose vocation may lead them to associate with, or to touch, or bury, the infected. For the rest, such persons as are not compelled to associate with the infected, may effectually avoid the contagion, however violent and deadly it may be, by avoiding contact. I am so perfectly convinced of this fact, from the experience and observation I have made during my residence at Mogodor, whilst the plague raged there in 1799, that I would not object to go to any country, although it were rotten with the plague, provided my going would benefit mankind, or serve any useful purpose; and I would use no fumigation, or any other remedy, but what I actually used at Mogodor in 1799, which I have detailed in the description of the plague, inserted in my account of Morocco, Timbuctoo, &c. I am so convinced, from my own repeated and daily experience, that the most deadly plague is as easy to be avoided *by strictly adhering to the principle of avoiding personal contact and inhalation, and the contact of infectious substances*, that I would ride or walk through the most populous and deeply-infected city, as I have done before, without any other precaution than a segar in my mouth; when, by avoiding contact and inhalation, I should most assuredly be free from the danger of infection!

When these precautions are strictly observed, I maintain, (in opposition to all the theoretical doctrines that have lately been propagated,) that there is no more danger of infection with the plague, than there is of infection from any common cold or rheum.—*Journal of Science and the Arts*, No. xv.

---

### *An Alphabetical List of the Plants and other Articles of the Materia Medica mentioned in the Works of* Hippocrates, *with the Latin Version of* Föes, *and the Synonyms of* Linnæus. [Extracted from a Memoir by Dr. Paulet.]

[*Continued from page* 300.]

Γαλβανον; *voyez* Χαλϐανον.

Γιγαρτα; o; *pépins de raisin.*

Γληχον; pulegium; mentha rotundifolia.

Γληχον χλωρον; polygonum viride; mentha pulegium, ou M. viridis?

---

* "M. Colaco having lately observed that oil was used externally to anoint the body, as a preservative against the plague, conceived the idea of administering this simple remedy *internally* to persons already infected. Numerous experiments were made by this gentleman; and, out of 300 individuals already infected, who resorted to this remedy, only twelve died."

Γλυκυρριζη; glycyrrhiza; glycyrrhiza glabra.

Γλυκυσις; pœonia; pœonia officinalis.

Γογγυλις; rapum; brassica rapa, ou B. oleracea

Δαις; tæda; *résine du* pinus pinea.

Δαφνη; laurus; laurus nobilis.

Δαφνοιδης; daphnoïdes; daphne laureola.

Δαυκος; daucus; buplevrum fruticosum?

Δαυκος κρητικη; daucus cretensis; athamanta cretensis.

Δαυκος αιθιοπικος; daucus æthiopicus; scandix odorata?

Δικταμνον; dictamnus; origanum heracleotium.

Δικταμνον κρητικον; dictamnum creticum; origanum dictamnus.

Δολιχος; phaseolus; phaseolus vulgaris.

Δρακοντιον; dracontium; arum dracunculus.

Δρυοπτερις; filix querna; polypodium dryopteris.

Εβενον; Ebenum; Diospyros ebenum.

Ηδυοσμος; mentha; mentha sativa.

Ελαια; olea; olea europæa.

Ελαια λευκη; oliva alba; *olive verte ou non mûre.*

Ελαιον αιγυπτιον λευκον; oleum ægyptium album; *huile du* sesamum orientale.

Ελατεριον; elaterium; *extrait du* momordica elaterium.

Ελελισφακος; salvia, mentha; salvia officinalis.

Ελενιον; helenium; hyssopus officinalis.

Ελλεβορος; veratrum; o.

Ελλεβορος μελας; veratrum nigrum; helleborus orientalis, LAMARCK.

Ελλεβορος λευκος; veratrum album; veratrum album.

Εγλεβορος μαλθαλος; veratrum molle; helleborus orientalis.

Ελξινη; elxine; polygonnm convolvulus? parietaria vulgaris? atractylis gummifera.

Εναvθεμον; enanthemum; adonis vernalis.

Επιπετρον; epipetrum; asplenium ruta muraria.

Επιθυμον et επιθυμον λευκον; epithymum album; cuscuta epithymum.

Ερεβινθος; cicer; cicer arietinum.

Ερευθεδανον; rubia; polygonum bistorta.

Ερικη; erica; erica vulgaris?

Ερπυλλος; serpillum; thymus serpillum.

Ερυον; ervum; ervum ervilia.

Ερυσιμον; erysimum, iris; erysimum vulgare.

Ερυθροδανον; rubia; rubia tinctorum.

Ετριγης; tragus, olyra; hordeum zeocrithon.

Ευσανθεμον; chamæmelum; achillea millefolium?

Ευζομον; eruca; brassica eruca.

Εχετροσις; bryonia alba; clematis vitalba.

Ζεα; siligo; triticum spelta.

Θαψια; thapsia, ferulago; thapsia asclepium *ou* garganica.

Θερμος; lupinum; lupinus albus.

Θλασπι; thlaspi; iberis amara.

Θλασπι λειον; thlaspi lævigatum; iberis semperflorens.

Θλασπι ποκιον; o; iberis umbellata.

Θριδαξ; lactuca; lactuca sativa.

Θυμβρα; thymbra; satureja thymbra *ou* S. hortensis.

Θυμον; thymum; satureja capitata.

Ινδικον; indicum; vitex negundo.

Ιξος; viscum; viscum album.

Ιον; viola; viola odorata.

Ιπποµαραθρον; hippomarathrum; cachrys sicula.

Ιπποφαη; hippophae; euphorbia spinosa??

Ιπποσελινον; ipposelinon, hipposelinum; pastinaca opopanax *ou* P. sylvestris.

Ιρις; iris; iris sambucina *et* I. florentina.

Ισαῖις; isatis; isatis tinctoria.

Ισχας; carica; *fruit sec du* ficus marisca.

Ισχεδιας μιχρος et μεγαλη; anchusa parva et magna, anchusa officinalis; angustifolia, lycopsis arvensis.

Ιλεα; salix; salix.

Καχρυς; cachrys, ros marinus, hordeum; imperatoria ostruthium, laserpitium latifolium, triticum turgidum, hordeum hexasticum.

Καλαμινθη; calamintha; melissa calamintha.

Καλαμος; calamus aromaticus; andropogon nardus.

Καλλιφυλλον; *voyez* αδιαντον.

Κανθια; canthia.

Καππαρις; capparis; capparis spinosa.

Καρδαμωμον; cardamomum; amomum cardamonum.

Καρδαμον; nasturtium et cardamum; lepidium sativum.

Καρδαμον αγριον; cardamum agreste; lepidium lyratum *ou* L. latifolium.

Καρυα ποντικα; nuces ponticæ; *fruits du* corylus avellana.

Καρυα ϛρογγυλα; nuces rotundæ; *fruits du* juglans regia.

Καρυον θασσιον; nux thasia; *fruits du* juglans fraxinifolia.

Κασιη; casia; amomi species ??

Καυκαλις; caucalis; cordylium syriacum.

Κηκις; galla; *galle du* quercus infectoria.

Κεδριδες; cedri baccæ; *fruits du* juniperus oxycedrus.

Κεδρος; cedrus; juniperus oxycedrus.

Κεδρος κρητικη; cedrus cretica; cedros cretica, TOURNEFORT.

Κεγχρος, κεγχρον; milium; panicum miliaceum.

Κεγχρυς et κεγχρυδας; milium, milii grana; *fruits des* panicum miliaceum et italicum.

Κενταυριον; centaurium; inula helenium, centaurea centaurium; gentiana lutea.

Κεϛρον; betonica; betonica officinalis.

Κινναμομον; cinnamomum; o.

Κισσος; hedera; hedera helix.

Κισθος ou κιϛος; cistus; cistus salvifolius *et* C. pilosus.

Κλεμαῖις; *voyez* εχιῖροσις.

Κνεκος; cnicus; carthamus tinctorius *et* C. cæruleus.

Κνεορον; cneorum; convolvulus cneorum, daphne alpina.

Κνεϛρον; cnestrum; daphne cneorum?

Κνικος; *voyez* κνεκος.

Κνιδη; urtica; urtica urens *et* U. dioïca.

Κοκκαλος; nux pinea; *pignon du* pinus pinea.

Κοκκος κνιδιος; granum cnidium; *fruit du* daphne gnidium.

Κοκκος; granum; *gallinsecte du* quercus coccifera?

Κολοκυνθη; cucurbita, colocynthis; cucurbita pepo.

Κολοκυνθις αγριη; cucurbita sylvestris; cucumis colocynthis.

Κομμι λευκον; gummi album; *gomme du* mimosa horrida.

Κωνειον; cicuta; conium maculatum.

Κονυζη; conyza; inula montana.

Κονυζη δισωδες; conyza fœtida; inula fœtida.

Κονυζη ηδυοσμος; conyza grati odoris; inula odora *ou* I. bifrons.

Κοριανον et κοριον; coriandrum; coriandrum sativum.

Κοτυληδον; umbilicus Veneris; cotyledon umbilicus Veneris.

Κραμβη; brassica; brassica.

Κρανιον; cornus *et* corna; *fruit du* cornus mas.

Κραταιγονον; *voyez* πολυκαρπον.

Κρεθμον; crethmon, crithmon; crithmum maritimum.

Κρινανθεμον; crinanthemum; lilium candidum.

